# B7-H5 blockade enhances CD8^+^ T-cell-mediated antitumor immunity in colorectal cancer

**DOI:** 10.1038/s41420-021-00628-4

**Published:** 2021-09-18

**Authors:** Jiayu Wang, Hongya Wu, Yanjun Chen, Jinghan Zhu, Linqing Sun, Juntao Li, Zhendong Yao, Yuqi Chen, Xueguang Zhang, Suhua Xia, Weichang Chen, Tongguo Shi

**Affiliations:** 1 Jiangsu Institute of Clinical Immunology, The First Affiliated Hospital of Soochow University, Suzhou, China; 2 Department of Gastroenterology, The First Affiliated Hospital of Soochow University, Suzhou, China; 3 Suzhou Key Laboratory for Tumor Immunology of Digestive Tract, The First Affiliated Hospital of Soochow University, Suzhou, China; 4 Jiangsu Key Laboratory of Clinical Immunology, Soochow University, Suzhou, China; 5 Jiangsu Key Laboratory of Gastrointestinal Tumor Immunology, The First Affiliated Hospital of Soochow University, Suzhou, China; 6 Department of Oncology, The First Affiliated Hospital of Soochow University, Suzhou, China

**Keywords:** Immunosuppression, Prognostic markers

## Abstract

Negative immune checkpoint blockade immunotherapy has shown potential for multiple malignancies including colorectal cancer (CRC). B7-H5, a novel negative immune checkpoint regulator, is highly expressed in tumor tissues and promotes tumor immune escape. However, the clinical significance of B7-H5 expression in CRC and the role of B7-H5 in the tumor microenvironment (TME) has not been fully clarified. In this study, we observed that high B7-H5 expression in CRC tissues was significantly correlated with the lymph node involvement, AJCC stage, and survival of CRC patients. A significant inverse correlation was also observed between B7-H5 expression and CD8^+^ T-cell infiltration in CRC tissues. Kaplan−Meier analysis showed that patients with high B7-H5 expression and low CD8^+^ T-cell infiltration had the worst prognosis in our cohort of CRC patients. Remarkably, both high B7-H5 expression and low CD8^+^ T infiltration were risk factors for overall survival. Additionally, B7-H5 blockade using a B7-H5 monoclonal antibody (B7-H5 mAb) effectively suppressed the growth of MC38 colon cancer tumors by enhancing the infiltration and Granzyme B production of CD8^+^ T cells. Importantly, the depletion of CD8^+^ T cells obviously abolished the antitumor effect of B7-H5 blockade in the MC38 tumors. In sum, our findings suggest that B7-H5 may be a valuably prognostic marker for CRC and a potential target for CRC immunotherapy.

## Introduction

Colorectal cancer (CRC) is a common malignant gastrointestinal tumor that seriously threatens human life and health [1]. Its morbidity and fatality rates have been on the rise in recent years. The global incidence and the mortality rate of CRC ranked third and second among all malignant tumors according to the statistics in 2020 [2]. The incidence of CRC is also increasing in China, especially in cities. Some traditional cancer treatments, such as surgery, chemotherapy, and radiation therapy, have been proven to prolong the 5-year life span of CRC patients, but these aforementioned treatments are still not ideal for CRC patients with advanced stages [3]. Therefore, it is extremely urgent to develop effective treatments for CRC patients and reduce the social burden caused by such diseases.

Immunotherapy based on negative immune checkpoint blockade has shown potential for multiple malignancies, such as melanoma, lung cancer, and CRC [4–6]. In 2017, the U.S. Food and Drug Administration (FDA) approved the monoclonal antibodies pembrolizumab and nivolumab against the immune checkpoint molecule PD-1 for second-line treatment of patients with CRC [5–7]. However, the efficacy of immune checkpoint blockade therapies in CRC patients is limited [8–10]. Therefore, it is meaningful to discover new immune checkpoints to enrich the treatment methods based on negative immune checkpoint blockade.

B7-H5, also known as VISTA, is one of the negative immune checkpoints of the B7 family, which was first discovered in 2011 [11]. B7-H5 is mainly expressed in myeloid cells such as macrophages, monocytes, dendritic cells, and T cells [12], and B7-H5 is highly expressed in a variety of tumors, including melanoma, pancreatic cancer, prostate cancer, non-small cell lung cancer, renal cell carcinoma, and CRC [13–17]. Importantly, the expression of B7-H5 has been associated with the prognosis of patients with CRC [15]. Isabelle et al. utilized a B7-H5-specific blocking mAb to demonstrate that B7-H5 blockade enhanced protective antitumor immunity and impaired the suppressive character of the tumor microenvironment (TME) [18]. In acute myeloid leukemia, myeloid-derived suppressor cells (MDSCs)-expressed B7-H5 significantly caused the inhibition of CD8^+^ T-cell activity [19]. These findings indicated that B7-H5 is an important negative checkpoint regulator in the TME, but the clinical significance of the B7-H5 expression in CRC and the role of B7-H5 in the TME remain to be elucidated.

In this study, the protein expression of B7-H5 was markedly increased in tumor tissues of CRC patients and associated with a poor prognosis of patients with CRC. In addition, there was a negative correlation between B7-H5 expression and CD8^+^ T-cell infiltration in CRC tissues. Moreover, we observed that B7-H5 blockade obviously suppressed tumor growth in mice bearing MC38 tumors by elevating the infiltration and Granzyme B production of CD8^+^ T cells.

## Results

### B7-H5 is highly expressed in CRC tissues and is correlated with the prognosis of CRC patients

**Fig. 1:**
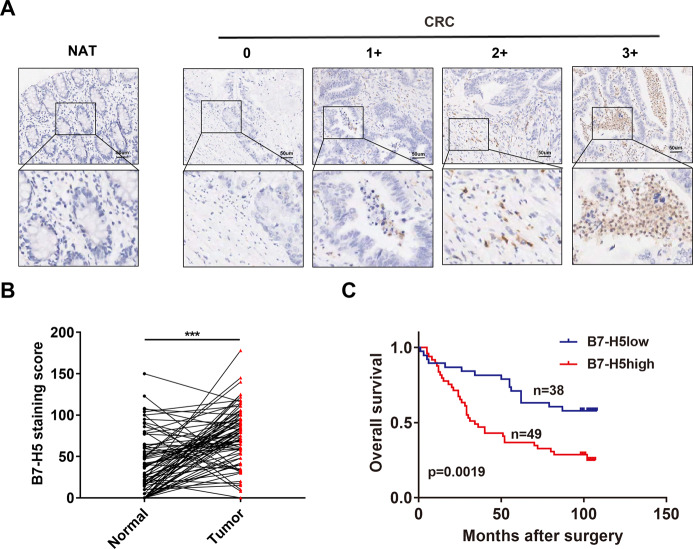
B7-H5 was abnormally expressed in CRC. **A** Representative B7-H5 IHC staining pattern of human CRC tissue and normal adjacent tissue (NAT). The staining score ranged from 0 to 3. **B** B7-H5 protein expression based on its staining index in CRC and NAT specimens. **C** The overall survival of patients with high or low B7-H5 expression was analyzed by Kaplan−Meier method (*P* = 0.0019). Values are expressed as means (SEMs). ***P < 0.001.

Here, an immunohistochemistry (IHC) assay was used to examine the protein expression of B7-H5 CRC tissues. As shown in Fig. 1A, the protein expression of B7-H5 was detected in immune cells of CRC tissues. B7-H5 expression was scored as 0, 1+, 2+, and 3+ according to the immunohistochemical expression intensity (Fig. 1A). We observed that 97.7% of tumor tissues showed B7-H5 expression and 2.3% did not express (Table 1). In addition, compared with normal adjacent tissues, the expression of B7-H5 in cancer tissues was significantly increased (Fig. 1B). Moreover, B7-H5 expression according to the clinical indicators of patients with CRC was shown in Table 1. The results noted that B7-H5 expression was significantly correlated with the lymph node involvement (*P* = 0.036), AJCC stage (*P* = 0.037), and survival (*P* = 0.026). Importantly, patients with high expression of B7-H5 (IHC score: 2+ and 3+) predicted a shorter overall survival compared with patients with low expression of B7-H5 (IHC score: 0 and 1+) (Fig. 1C).

**Table 1:** B7-H5 expression and clinical features in 87 colorectal cancer patient samples.

Characteristic	Total no.	B7-H5 expression				*P* value
		0	1	2	3	
All cases	87	2	36	27	22	
Age						
<70	37	0	18	11	8	
≥70	50	2	18	16	14	
Gender						0.292
Male	46	1	20	17	8	
Female	41	1	16	10	14	
Tumor depth						0.243
T1/T2	6	0	1	3	2	
T3/T4	81	2	35	24	20	
LN involvement						0.036
N0	53	1	26	18	9	
N1/N2	34	1	10	11	13	
Pathological grading						0.51
Moderate	48	1	23	11	13	
Poor	39	1	13	18	9	
AJCC stage						0.037
I/II	53	1	26	17	9	
III/IV	34	1	10	10	13	
Death						0.026
−	35	0	22	6	7	
+	52	2	14	21	15	

### B7-H5 expression and CD8^+^ T infiltration in CRC tissues

**Fig. 2:**
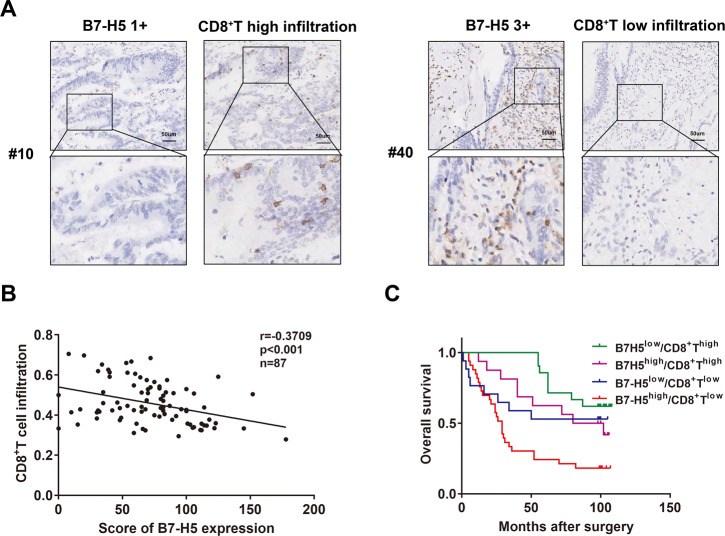
Relationship between B7-H5 and CD8^+^ T-cell infiltration in CRC. **A** Representative B7-H5 and CD8^+^ T IHC staining images from patients #10 and #40 were shown. **B** The correlation between B7-H5 expression and CD8^+^ T-cell infiltration was analyzed. *r* = −0.3709, *P* < 0.001. **C** Kaplan−Meier curves of overall survival of CRC patients according to four subtypes based on B7-H5 and CD8 expression.

Next, we examined the relationship between B7-H5 expression and CD8^+^ T-cell infiltration in tissue samples of patients with CRC. As shown in Fig. 2A, B, there was a markedly negative correlation between B7-H5 expression and CD8^+^ T-cell infiltration in CRC tissue (*r* = −0.3709, *P* < 0.001). Moreover, the prognostic value of B7-H5 expression and CD8^+^ T infiltration in CRC tissue was evaluated. As shown in Fig. 2C, we classified four types of B7-H5/CD8 expression, namely, B7-H5^low^CD8^+^T^low^, B7-H5^low^CD8^+^T^high^, B7-H5^high^CD8^+^T^low^, and B7-H5^high^CD8^+^T^high^. The results of the Kaplan−Meier survival analysis showed that patients in the B7-H5^high^CD8^+^T^low^ group had a significantly worse prognosis than those in the other three groups (Fig. 2C). In addition, univariate and multivariate analysis indicated that age, N stage, high level of B7-H5 expression, and low levels of CD8^+^ T infiltration were risk factors for the overall survival of patients with CRC (Table 2).

**Table 2:** Univariate and multivariate analysis of overall survival.

Characteristic	Univariate	*P* value	Characteristic	Multivariate	*P* value
	HR (95% CI)			HR (95% CI)	
Gender			Gender		
Male vs female	1.022 (0.592–1.764)	0.937	Male vs female	0.96 (0.503–1.835)	0.903
Age			Age		
<70 vs ≥70	2.122 (1.175–3.833)	0.013	<70 vs ≥70	1.932 (1.008–3.704)	0.047
T stage			T stage		
T1/T2 vs T3/T4	2.42 (0.588–9.958)	0.221	T1/T2 vs T3/T4	2.047 (0.476–8.805)	0.336
N stage			N stage		
N0 vs N1/N2	2.847 (1.638–4.949)	0.0002	N0 vs N1/N2	2.391 (1.272–4.493)	0.007
B7-H5 expression			B7-H5 expression		
Low vs high	2.479 (1.369–4.488)	0.003	Low vs high	1.984 (1.058–3.718)	0.033
CD8 infiltration			CD8 infiltration		
Low vs high	0.387 (0.215–0.696)	0.002	Low vs high	0.478 (0.258–0.888)	0.019
Pathological grading			Pathological grading		
Moderate vs poor	1.506 (0.874–2.597)	0.14	Moderate vs poor	1.54 (0.854–2.775)	0.151

### B7-H5 inhibition suppresses tumor growth and promotes CD8^+^ T-cell infiltration

**Fig. 3:**
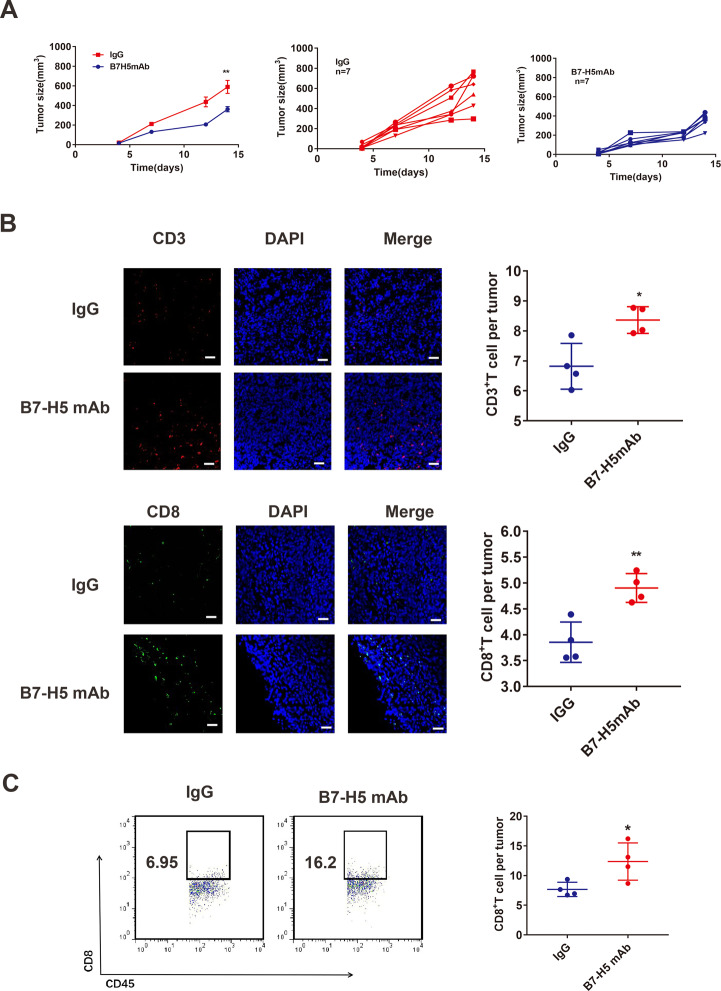
B7-H5 inhibition suppressed tumor growth in mice and promoted CD8^+^ T-cell infiltration in the tumor tissues of MC38 model. **A** Growth curve of MC38 tumors after treatment with monoclonal anti-mouse B7-H5 antibody (B7-H5 mAb) or control IgG. *n* = 7. The experiments were performed in triplicate. Values are expressed as means (SEMs). **P < 0.01. **B** The proportion of CD3^+^ T and CD8^+^ T cells in tumor tissues from MC38 tumors after treatment with B7-H5 mAb or control IgG was analyzed by immunofluorescence (original magnification ×200). A representative image was shown. *n* = 4. The experiments were performed in triplicate. Values are expressed as means (SD). *P < 0.05; **P < 0.01. **C** The proportion of CD8^+^ T cells in the CD45^+^ T cells of tumor tissues from MC38 tumors after treatment with B7-H5 mAb or control IgG was analyzed by flow cytometry. A representative image was shown. *n* = 4. The experiments were performed in triplicate.

To investigate the role of B7-H5 in CRC tumors, MC38 mouse tumor models were established and administered B7-H5 mAb. As shown in Fig. 3A, treatment with B7-H5 mAb significantly suppressed the growth of MC38 tumors.

Then, we investigated whether the therapeutic effects of B7-H5 mAb were associated with CD8^+^ T cells. The sections from MC38 tumors were firstly evaluated for hematoxylin and eosin (H&E) staining (Supplementary Fig. 1). The results of immunofluorescence staining indicated that B7-H5 mAb treatment obviously enhanced the immune cell infiltration, evidenced by the ratio of CD3^+^ T cells in the MC38 tumor tissues (Fig. 3B). Importantly, compared with the control group, the ratio of CD8^+^ T cells was significantly increased in the tumor tissues of the B7-H5 mAb treatment group (Fig. 3B). In addition, we obtained similar results by flow cytometry assay (Fig. 3C).

### B7-H5 blockade-mediated tumor growth inhibition is CD8^+^ T-cell-dependent

**Fig. 4:**
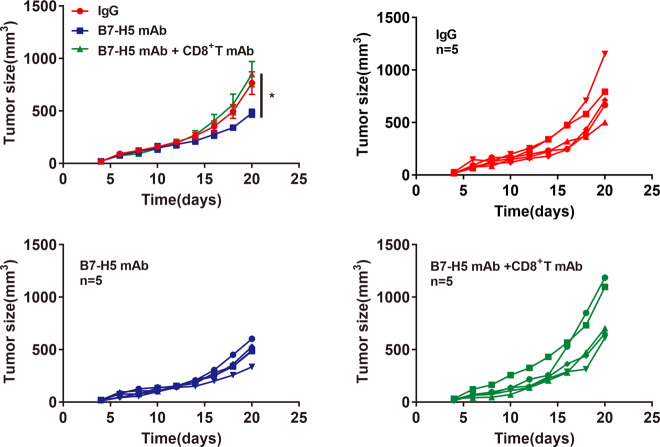
B7-H5 blockade-mediated tumor growth inhibition was CD8^+^ T-cell dependent. Growth curve of MC38 tumors after co-treated with B7-H5 mAb and monoclonal anti-mouse CD8 (CD8^+^ T mAb). *n* = 5. The experiments were performed in triplicate. Values are expressed as means (SD). *P < 0.05.

Having demonstrated that B7-H5 inhibition increased the infiltration of CD8^+^ T cells, we next sought to examine whether the therapeutic effects of B7-H5 blockade were CD8^+^ T-cell-dependent. As shown in Fig. 4, the depletion of CD8^+^ T cells obviously abolished the antitumor effect of anti-B7-H5 blockade alone.

### B7-H5 blockade promotes Granzyme B expression by CD8^+^ T cells

**Fig. 5:**
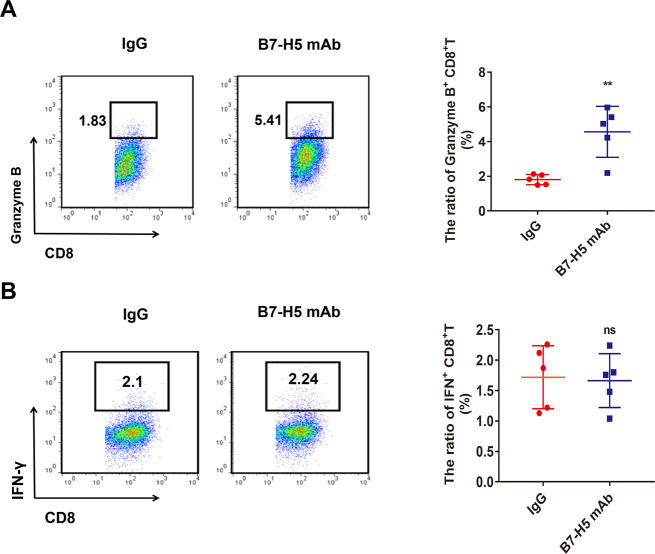
B7-H5 blockade affects the cytokine expression of CD8^+^ T cells in vitro. **A**, **B** The intracellular Granzyme B (A) and IFN-γ (B) expression in CD8^+^ T cells after treatment with IgG or B7-H5 mAb were detected by flow cytometry. A representative image is shown. The experiments were performed in triplicate. Values are expressed as means (SD). **P < 0.01.

Next, we sought to explore the role of B7-H5 in modulating the function of CD8^+^ T cells in vitro. Intracellular Granzyme B and IFN-γ levels in CD8^+^ T cells after treatment with or without B7-H5 mAb were analyzed by flow cytometry. Compared with the control group, the proportion of Granzyme B-positive CD8^+^ T cells was significantly increased in the B7-H5 mAb group (Fig. 5A). However, B7-H5 inhibition had no effect on the proportion of IFN-γ positive CD8^+^ T cells (Fig. 5B).

## Discussion

It has been previously reported that the aberrant expression of B7-H5 in cancers is related to the poor prognosis for multiple cancers, such as lung cancer, oral squamous cell carcinoma, and CRC [20]. Franz et al. showed that elevated expression of B7-H5 was significantly associated with longer 5-year overall survival of patients with non-small cell lung cancer [14]. In human oral squamous cell carcinoma, the B7-H5 protein expression was markedly increased in tumor tissues and was correlated with lymph node status [21]. Herein, we observed that compared with normal adjacent tissues, the expression of B7-H5 was significantly increased in cancer tissues. Moreover, B7-H5 expression was significantly correlated with the lymph node involvement, AJCC stage, and survival in our cohort of CRC patients. These results were consistent with a previous study, which also indicated that high expression of B7-H5 mRNA was associated with poor prognosis in colon cancer in a cohort of patients from The Cancer Genome Atlas (TCGA) dataset GSE40967 [22]. The study by Shan et al. found that the protein levels of B7-H5 in CRC specimens were much higher than those in para-tumors and normal tissues [15]. Another discrepancy report showed that high B7-H5 expression was observed significantly more frequently in patients with N0 stage, stage I–II, and colon adenocarcinoma, and correlated with a favorable prognosis [23]. The inconsistent data on the correlation between high B7-H5 expression and prognosis of CRC patients might be related to the CRC specimens with different stages. In the current study, the univariate and multivariate analysis also showed that abnormal B7-H5 expression increases the risk of death in patients. Thus, high B7-H5 expression in CRC can act as a prognostic factor.

Tumor-infiltrating CD8^+^ T cells are correlated with better patient prognosis in various tumor types including CRC [24]. For example, ovarian cancer patients with more tumor-infiltrating CD8^+^ T lymphocytes had a significantly better prognosis compared with patients with less count [25]. In addition, tumor-infiltrating CD8^+^ T cells were an independent positive prognostic marker in CRC [26]. Hence, we further analyzed the correlation between B7-H5 expression and CD8^+^ T-cell infiltration in cancer lesions in our cohort of CRC patients. A significant inverse correlation was observed between B7-H5 expression and CD8^+^ T-cell infiltration in CRC tissues. Interestingly, the survival analysis of B7-H5 expression in combination with CD8^+^ T-cell infiltration in CRC lesions showed that patients with high B7-H5 expression and low CD8^+^ T-cell infiltration had the worst prognosis in our cohort of CRC patients. Besides, both high levels of B7-H5 expression and low levels of CD8^+^ T infiltration were risk factors for the overall survival of patients with CRC. These results suggest that B7-H5 expression synergized with CD8^+^ T-cell infiltration may be a valuably clinical prognostic parameter for CRC. However, the relationship between B7-H5 and other immune cells such as CD4^+^ T cells, tumor-associated cells (TAMs), and MDSCs in the CRC TME should be further clarified.

B7-H5 has been demonstrated to be an important negative regulator of T-cell function, which further mediated the immune escape of several malignancies such as melanoma, bladder, glioma, and CRC [18, 27, 28]. Isabelle et al. noted that B7-H5 mAb treatment not only elevated the infiltration, proliferation, and effector function of tumor-reactive T cells in the TME in both melanoma and bladder mouse models but also altered the suppressive feature of the TME [18]. Moreover, treatment with B7-H5 mAb effectively impaired the growth of established melanoma or bladder tumors [18, 29]. B7-H5 knockout mice were highly resistant to tumor induction in a murine brain glioma model through activating CD4^+^ T-cell-mediated immunity [27]. Additionally, a blockade using a monoclonal antibody specific for B7-H5 led to tumor regression in the CT26 colon cancer model [28]. In the present study, we observed that B7-H5 inhibition using a commercial monoclonal antibody effectively suppressed the growth of MC38 colon cancer tumors. Furthermore, B7-H5 mAb administration significantly increased CD8^+^ T-cell infiltration in the MC38 tumor tissues. More importantly, depletion of CD8^+^ T cells obviously abolished the antitumor effect of anti-B7-H5 blockade in the MC38 colon cancer tumors. Besides, B7-H5 has been found to inhibit mouse and human CD8^+^ T-cell proliferation, as well as the production of cytokines [11]. Herein, our results showed that B7-H5 inhibition significantly increased the proportion of Granzyme B-positive CD8^+^ T cells in vitro. These results suggest that B7-H5 blockade-mediated tumor regression in the MC38 colon cancer model in a CD8^+^ T-cell-dependent manner. However, the precise role and mechanism of B7-H5 in the regulation of CD8^+^ T-cell infiltration or cytokine production in the TME of CRC is still unclear. Further investigations are required to answer this question.

In conclusion, our study showed that B7-H5 was highly expressed in CRC, which was closely related to the prognosis of patients. Moreover, B7-H5 expression synergized with CD8^+^ T-cell infiltration may predict the survival of CRC. Importantly, our results revealed that B7-H5 blockade inhibited tumor growth by enhancing CD8^+^ T-cell infiltration and response in the TME of CRC. Collectively, B7-H5 may be a valuably prognostic marker for CRC and a potential target for CRC immunotherapy.

## Materials and methods

### Patients and specimens

CRC tissue microarray (TMA) chips were obtained from Shanghai Biochip Co., Ltd. (Shanghai, China), which contained 87 pairs of CRC tissue samples and adjacent normal tissues with follow-up data. The relevant clinical data of each patient were collected and provided in Table 1. Overall survival was defined as the time elapsed from surgery to death. Also, we obtained approval for this study from the Ethics Review Board of the First Affiliated Hospital of Soochow University.

### Immunohistochemistry

IHC was performed as previously described [30]. In brief, paraffin-embedded tissues in the CRC TMA chip or mouse tumor tissues were incubated with rabbit anti-human VISTA (B7-H5) antibody (CST, Danvers, MA, USA) and rabbit anti-human CD8α antibody (Abcam, Cambridge, MA, USA) at 4 °C overnight, and this was followed by incubation with the corresponding HRP-labeled secondary antibody (Genetech, China) for 45 min at room temperature.

The score of IHC was judged by two experienced pathologists. About the B7-H5 staining score, the rating intensity was 0−3 points (0, no staining; 1+, low staining; 2+, moderate staining; 3+, high staining). To facilitate research, samples were divided into the low expression group (with scores of 0 and 1) and the high expression group (with scores of 2 and 3). Each organization area was scored according to formula (3+ percent cells) × 3+ (2+ percent cells) × 2+ (1+ percent cells) × 1. Then an immunohistochemistry score ranging from 0 to 300 was produced. The ratio of the number of positive cells to the total number of positive cells in the tumor (between 0 and 1) is regarded as the evaluation of CD8+ T infiltration.

### Cell culture

MC38 cells were purchased from the American Type Culture Collection (ATCC, Manassas, VA), and were cultured in Dulbecco’s Modified Eagle Medium (DMEM, Biological Industries, BeitHaemek, Israel) supplemented with 10% fetal bovine serum (Biological Industries) and 1% penicillin-streptomycin (Beyotime, Shanghai, China).

### Mouse tumor experiments

C57BL/6 female mice aged 6−8 weeks were purchased from Laboratory Animal Technology Co., Ltd (Shanghai, China). MC38 cells (1 × 10^6^ per mouse) were subcutaneously injected into the right flanks of C57BL/6 mice. To evaluate the effect of B7-H5 on tumor progression, tumor-bearing mice were intraperitoneally injected with monoclonal anti-mouse B7-H5 antibody (B7-H5 mAb, BioXCell, West Lebanon, NH, USA) or control IgG at a dose of 200 µg/mouse twice a week. To investigate whether the therapeutic effect of B7-H5 mAb was CD8^+^ T-cell-dependent, tumor-bearing mice were co-treated with B7-H5 mAb and monoclonal anti-mouse CD8 (CD8^+^ T mAb, BioXCell) at a dose of 200 µg/mouse through intraperitoneal injection twice per week. Tumor size was monitored every 2–3 days. The tumor volume was calculated according to the formula: 1/2 × L(length) × W(width) [2]. For H&E staining, frozen sections from the xenografted tumor tissues were fixed with 4% paraformaldehyde, stained by H&E kit (Beyotime, Shanghai, China) according to the manufacturer’s protocol.

### Immunofluorescent analysis

Frozen sections from mouse tumor tissues (thickness 5 µm) were blocked with 5% bovine serum albumin for 1 h at 37 °C and stained with anti-mouse CD3 antibody (Proteintech, Wuhan, China) or FITC-conjugated anti-mouse CD8 antibody (Biolegend, San Diego, CA, USA) overnight at 4 °C. For CD3 staining, the sections were incubated with Alexa Fluor® 594-labeled goat anti-rabbit secondary antibody (Abcam) staining for 45 min at room temperature. Next, the slides were incubated with DAPI at room temperature for 5 min. All fluorescence images were taken using a Nikon Eclipse/ NI-U microscope. The CD3^+^ T and CD8^+^ T ratio was calculated in three random view fields.

### Flow cytometry

The tumor tissue of the mouse was digested with DNaseI (0.02 mg/ml, Sigma-Aldrich, St. Louis, MO, USA) and LiberaseTL (0.2 mg/ml, Roche, Basel, Switzerland) in serum-free RPMI1640 medium for 30 min. After filtered using a 70 µm cell filter, single-cell suspensions were further stained with the following fluorochrome-conjugated antibodies: PE-conjugated anti-CD45 (Biolegend) and FITC-conjugated anti-CD8 (Biolegend).

### CD8^+^ T-cell isolation

CD8+ T cells were isolated from the spleen of BALB/c mice using anti-PE MicroBeads (Miltenyi Biotec) and PE-conjugated anti-CD8 (Biolegend) according to the manufacturer’s protocol.

### Intracellular IFN-γ and Granzyme B staining

For intracellular IFN-γ and Granzyme B staining, CD8^+^ T cells (2 × 10^5^ cells per well) were seeded in 24-well plates and treated with or without anti-CD3, anti-CD28 (Biolegend), or monoclonal anti-mouse B7-H5 antibody. After 72 h, CD8^+^ T cells were incubated with Brefeldin A (Biolegend) for 10 h. Subsequently, cells were surface stained with PE-conjugated anti-CD8 (Biolegend), followed by intracellular staining for APC-conjugated anti-IFN-γ (Invitrogen, Carlsbad, CA, USA) and PE/Cy7-conjugated anti-Granzyme B (Biolegend).

### Statistical analysis

Statistical analysis was performed using the SPSS software (IBM Corporation, Armonk, NY, USA). A chi-square test was used to compare the expression of B7-H5 protein. Pearson correlation analysis was used to analyze the correlation between B7-H5 and CD8^+^ T infiltration. Survival analysis was performed by using the Kaplan–Meier method. *P* < 0.05 was considered statistically significant.

## Supplementary information

Supplementary fig.1

Author contribution statement

## Data Availability

The datasets used and analyzed during the current study are available from the corresponding authors on reasonable request.

## References

[1] Siegel R, Miller K, Jemal A (2019). Cancer statistics, 2019. CA: Cancer J. Clin.

[2] Sung, H, Ferlay J, Siegel RL, Laversanne M, Soerjomataram I, Jemal A. Global cancer statistics 2020: GLOBOCAN estimates of incidence and mortality worldwide for 36 cancers in 185 countries. CA: Cancer J Clin. 2021. 10.3322/caac.21660 10.3322/caac.21660 33538338

[3] Dekker E, Tanis P, Vleugels J, Kasi P, Wallace M (2019). Colorectal cancer. Lancet.

[4] Hodi FS, Lawrence D, Lezcano C, Wu X, Zhou J, Sasada T (2014). Bevacizumab plus ipilimumab in patients with metastatic melanoma. Cancer Immunol Res.

[5] Le DT, Durham JN, Smith KN, Wang H, Bartlett BR, Aulakh LK (2017). Mismatch repair deficiency predicts response of solid tumors to PD-1 blockade. Science.

[6] Hendriks L, Besse B (2018). New windows open for immunotherapy in lung cancer. Nature.

[7] Overman MJ, McDermott R, Leach JL, Lonardi S, Lenz HJ, Morse MA (2017). Nivolumab in patients with metastatic DNA mismatch repair-deficient or microsatellite instability-high colorectal cancer (CheckMate 142): an open-label, multicentre, phase 2 study. Lancet Oncol.

[8] Zou W, Wolchok J, Chen L (2016). PD-L1 (B7-H1) and PD-1 pathway blockade for cancer therapy: mechanisms, response biomarkers, and combinations. Sci Transl Med.

[9] de Miguel M, Calvo E (2020). Clinical challenges of immune checkpoint inhibitors. Cancer Cell.

[10] Kalbasi A, Ribas A (2020). Tumour-intrinsic resistance to immune checkpoint blockade. Nat Rev Immunol.

[11] Wang L, Rubinstein R, Lines JL, Wasiuk A, Ahonen C, Guo Y (2011). VISTA, a novel mouse Ig superfamily ligand that negatively regulates T cell responses. J Exp Med.

[12] Ni L, Dong C (2017). New checkpoints in cancer immunotherapy. Immunol Rev.

[13] Gao J, Ward JF, Pettaway CA, Shi LZ, Subudhi SK, Vence LM (2017). VISTA is an inhibitory immune checkpoint that is increased after ipilimumab therapy in patients with prostate cancer. Nat Med.

[14] Villarroel-Espindola F, Yu X, Datar I, Mani N, Sanmamed M, Velcheti V (2018). Spatially resolved and quantitative analysis of VISTA/PD-1H as a novel immunotherapy target in human non-small cell lung cancer. Clin Cancer Res.

[15] Xie S, Huang J, Qiao Q, Zang W, Hong S, Tan H (2018). Expression of the inhibitory B7 family molecule VISTA in human colorectal carcinoma tumors. Cancer Immunol Immunother.

[16] Blando J, Sharma A, Higa MG, Zhao H, Vence L, Yadav SS (2019). Comparison of immune infiltrates in melanoma and pancreatic cancer highlights VISTA as a potential target in pancreatic cancer. Proc Natl Acad Sci USA.

[17] Hong S, Yuan Q, Xia H, Zhu G, Feng Y, Wang Q (2019). Analysis of VISTA expression and function in renal cell carcinoma highlights VISTA as a potential target for immunotherapy. Protein Cell.

[18] Le Mercier I, Chen W, Lines JL, Day M, Li J, Sergent P (2014). VISTA regulates the development of protective antitumor immunity. Cancer Res.

[19] Wang L, Jia B, Claxton DF, Ehmann WC, Rybka WB, Mineishi S (2018). VISTA is highly expressed on MDSCs and mediates an inhibition of T cell response in patients with AML. Oncoimmunology.

[20] Zhong C, Lang Q, Yu J, Wu S, Xu F, Tian Y (2020). Phenotypical and potential functional characteristics of different immune cells expressing CD28H/B7-H5 and their relationship with cancer prognosis. Clin Exp Immunol.

[21] Wu L, Deng WW, Huang CF, Bu LL, Yu GT, Mao L (2017). Expression of VISTA correlated with immunosuppression and synergized with CD8 to predict survival in human oral squamous cell carcinoma. Cancer Immunol Immunother.

[22] Deng J, Li J, Sarde A, Lines JL, Lee YC, Qian DC (2019). Hypoxia-induced VISTA promotes the suppressive function of myeloid-derived suppressor cells in the tumor microenvironment. Cancer Immunol Res.

[23] Zong L, Yu S, Mo S, Zhou Y, Xiang Y, Lu Z (2021). Expression correlates with a favorable prognosis in patients with colorectal cancer. J Immunother.

[24] van der Leun A, Thommen D, Schumacher T (2020). CD8 T cell states in human cancer: insights from single-cell analysis. Nat Rev Cancer.

[25] Hamanishi J, Mandai M, Iwasaki M, Okazaki T, Tanaka Y, Yamaguchi K (2007). Programmed cell death 1 ligand 1 and tumor-infiltrating CD8^+^ T lymphocytes are prognostic factors of human ovarian cancer. Proc Natl Acad Sci USA.

[26] Pagès F, Berger A, Camus M, Sanchez-Cabo F, Costes A, Molidor R (2005). Effector memory T cells, early metastasis, and survival in colorectal cancer. N Engl J Med.

[27] Flies DB, Han X, Higuchi T, Zheng L, Sun J, Ye JJ, Chen L (2014). Coinhibitory receptor PD-1H preferentially suppresses CD4 T cell-mediated immunity. J Clin Investig.

[28] Liu J, Yuan Y, Chen W, Putra J, Suriawinata AA, Schenk AD (2015). Immune-checkpoint proteins VISTA and PD-1 nonredundantly regulate murine T-cell responses. Proc Natl Acad Sci USA.

[29] Xu W, Dong J, Zheng Y, Zhou J, Yuan Y, Ta HM (2019). Immune-checkpoint protein VISTA regulates antitumor immunity by controlling myeloid cell-mediated inflammation and immunosuppression. Cancer Immunol Res.

[30] Ding S, Lv X, Liu Z, Zhan S, Xu Y, Zhang X (2021). Overexpression of B7-H4 is associated with infiltrating immune cells and poor prognosis in metastatic colorectal cancer. Int Immunopharmacol.

