# Supplementary material for: B7-H5 blockade enhances CD8+ T-cell-mediated antitumor immunity in colorectal cancer
Source: Cell Death Discov. 2021 Sep 18;7:248. doi: 10.1038/s41420-021-00628-4 (PMC8449782; doi:10.1038/s41420-021-00628-4)
Supplement: Supplementary file 1 — Supplementary fig.1 [file 41420_2021_628_MOESM1_ESM.docx]

Supplementary figure 1


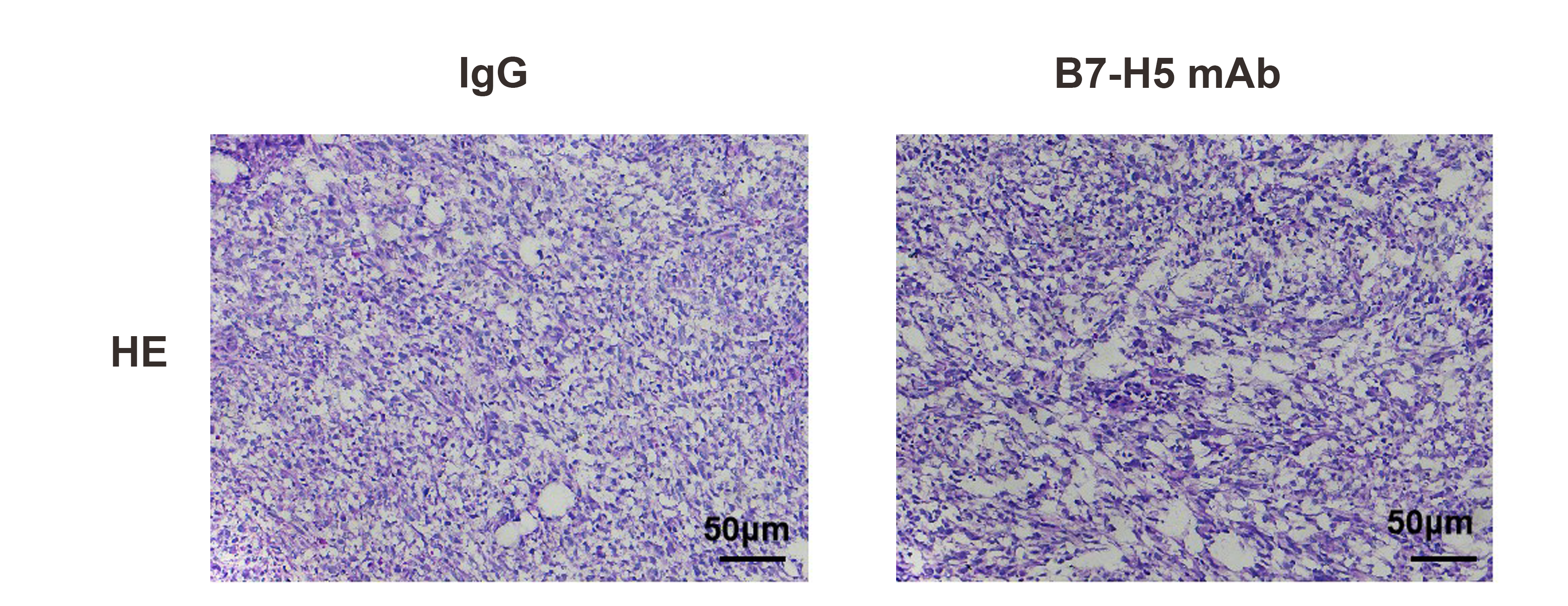


**Figure S1. Representative pictures of H&E staining**

Representative H&E staining images of tumor sections from the IgG or B7-H5 mAb group. (original magnification ×200).
