# Supplementary material for: B7-H5 blockade enhances CD8+ T-cell-mediated antitumor immunity in colorectal cancer
Source: Cell Death Discov. 2021 Sep 18;7:248. doi: 10.1038/s41420-021-00628-4 (PMC8449782; doi:10.1038/s41420-021-00628-4)
Supplement: Supplementary file 2 — Author contribution statement [file 41420_2021_628_MOESM2_ESM.docx]

A**uthor contribution statement**

1. Jiayu Wang and Hongya Wu: Methodology; Formal Analysis; Writing – Original Draft
2. Yanjun Chen: Writing – Review & Editing;
3. Jinghan Zhu: Complete part of the experiment
4. Linqing Sun: Complete part of the experiment
5. Juntao Li: Complete part of the experiment
6. Zhendong Yao: Complete part of the experiment
7. Yuqi Chen: Complete part of the experiment
8. Xueguang Zhang: Complete part of the experiment
9. Suhua Xia: Conceive and design experiments
10. Weichang Chen: Conceive and design experiments; Approved the final version
11. Tongguo Shi: Conceive and design experiments; Funding Acquisition
